# Antimicrobial activity of *Terminalia catappa* brown leaf extracts against *Staphylococcus aureus* ATCC 25923 and *Pseudomonas aeruginosa* ATCC 27853

**DOI:** 10.12688/f1000research.15998.1

**Published:** 2018-09-04

**Authors:** Ovin Qonita Allyn, Eko Kusumawati, Rudy Agung Nugroho

**Affiliations:** 1 Animal Physiology, Development, and Molecular Laboratory, Department of Biology, Faculty of Mathematics and Natural Sciences, Mulawarman University, Samarinda, Kalimantan Timur, 75123, Indonesia; 2 Microbiology and Genetic Molecular Laboratory, Department of Biology, Faculty of Mathematics and Natural Sciences, Mulawarman University, Samarinda, Kalimantan Timur, 75123, Indonesia

**Keywords:** Terminalia catappa, phytochemicals, Staphylococcus aureus, Pseudomonas aeruginosa, antibacterial

## Abstract

The aim of this study was to determine the effects of various concentration of *Terminalia catappa* brown leaves extract which can inhibit the growth of *Staphylococcus aureus* ATCC 25923 and *Pseudomonas aeruginosa *ATCC 27853. The crushed-brown leaves of *Terminalia catappa* was extracted using 95% ethanol, filtered, and evaporated. The dried *T. catappa* extract was used to identify phytochemical content qualitatively. Total phenolic and flavonoid contents were also measured quantitatively from dried extract. The dried extracts were also dissolved in sterile aquadest and serial dilutions were prepared to final concentration of 30, 60 and 90%. A disc diffusion method was used to evaluate the antibacterial activity of various concentrations of ethanol extract of brown leaves of *T. catappa*. Inhibition zone diameter was measured to determine antibacterial activity. Gentamycin sulfate and distilled water were used as positive and negative controls, respectively. Dried ethanolic extract of brown *T. catappa* leaves contained flavonoid, quinon, phenolic, triterpenoid, and tannin. A total of 208.722 mg gallic acid equivalent/g extract of total phenolic and 35.7671 mg quercetin equivalent/g extract of total flavonoid were also found in the dried extract. The inhibition zone diameters of ethanolic extracts ranged from 1.73 to 9.06 mm ( *S. aureus*) and from 1.83 to 6.5 mm ( *P. aeruginosa*). The higher concentration of extract, the wider the inhibition zone diameters for both bacteria. *P. aeruginosa* was more resistant to high concentrations of extract (90%) than *S. aureus*. Ethanolic extracts of the brown leaves of *T. catappa* had different antibacterial effects against *S. aureus* and *P. aeruginosa*. The higher the concentration of extract, the wider the inhibition zone diameter for both bacteria. *P. aeruginosa* was more resistant to high concentrations of ethanolic extracts of the brown leaves of *T. catappa*.

## Introduction

*Staphylococcus* and *Pseudomonas* species have been identified as causative agents of disease and serious pathogens in many aquatic animals, including fish ^1– 4^, resulting in high mortality rates in many commercially farmed fish. Among various *Staphylococcus* and *Pseudomonas* species, *Staphylococcus aureus* and *Pseudomonas aeruginosa* are known to cause disease in *Oreochromis niloticus* and *Oreochromis mossambicus* ^5^. To reduce high mortality rates in farmed fish, aquaculturists and researcher used chemical agents and antibiotics to promote growth or prevent *S. aureus* and *P. aeruginosa* infection ^6^.

However, the use of antibiotics to prevent and cure common infectious diseases in fish is becoming increasingly limited due to environmental concern, and increasingly expensive and ineffective because of microbial resistance ^7– 9^. As alternatives, various plant extracts, such as those of *Boesenbergia pandurata*, *Zingiber zerumbet* and *Solanum ferox*, have been tested and used as an alternative to antibiotics ^10– 12^. Another potential plant extract that can be used as an antimicrobial is that of *Terminalia catappa*, which is widely distributed in tropical and sub-tropical regions, including Indonesia ^13, 14^.

*Terminalia catappa* L., belonging to the family Combretaceae, is a large deciduous tree. The aqueous extract of *Terminalia catappa* leaves has been known as a folk medicine for antipyretic, hemostatic, hepatitis and liver-related diseases purposes in the Philippines, Malaysia and Indonesia ^15, 16^. Past research revealed that the extract of *T. catappa* leaves can be used to improve a resistance to *Aeromonas hydrophila* in *Betta* sp ^17^, remedy against tilapia ( *Oreochromis niloticus*) parasites and bacterial pathogen ^18, 19^. Nevertheless, scientific literature concerning the antibacterial potency of *T. catappa* against *Staphylococcus aureus* and *Pseudomonas aeruginosa* is limited.

Thus, the aim of the study was to evaluate the effects of various concentration of *T. catappa* brown leaf extract on the growth of *Staphylococcus aureus* and *Pseudomonas aeruginosa* by calculating inhibition zone diameters. The phytochemical content of the extract was also qualitatively determined and the flavonoid and phenolic concentrations in the extract was quantified.

## Methods

### Site and time

The research was performed from March to May 2018 at the Animal Physiology, Development and Molecular Laboratory for extracting *T. catappa* leaves. Meanwhile, assay study was done at microbiology and molecular genetic laboratory.

### Bacterial strains and culture condition

Bacterial strains were obtained from a Microbiology Laboratory, Faculty of Pharmacy, Sumatera Utara University, Indonesia. *Staphylococcus aureus* ATCC 25923 and *Pseudomonas aeruginosa* ATCC 27853 were used to investigate the antibacterial activity. Both bacteria were sub-cultured on nutrient agar and stored at 4°C until use.

### Plant materials

Brown leaves of *T. catappa* were collected from a region of Mulawarman university campus, Samarinda, East Kalimantan. Leaves were dried at room temperature for 2 days, crushed, transferred into a glass container and preserved until the extraction procedure.

### Extraction procedure

Approximately 1 kg of crushed leaves was soaked in 1 l of 95% ethanol for 5 days and shaken occasionally with a shaker. After 5 days, materials were filtered (Whatman No. 11 paper filter). The filtrate was evaporated using a rotary evaporator. Finally, the dried extracts were obtained and stored at 4°C in a dark bottle until use. The dried extracts were then dissolved in sterile distilled water and serial dilutions were prepared to give final concentrations of 30, 60 and 90%.

### Phytochemical content

Dried extract samples were subjected to qualitative phytochemical analysis for flavonoids, quinon, alkaloids, phenolic, steroid, triterpenoid, saponins, and tannins using standard methods as previously described by Nugroho *et al*. ^20^. Meanwhile, total phenolics and flavonoids were quantitatively measured, using the method described by Pourmorad *et al*. ^21^.

### Antibacterial activity assay

The antibacterial activity of *T. catappa* brown leaf ethanolic extract was evaluated using the disc diffusion method ^22^. Three replicated agar plates were used for each different concentration and both controls (distilled water and 0.1% gentamycin sulfate). A total of 10 µl extract was added to a paper disc for each concentration and controls. Each disk was then placed in agar plate which had bacterial suspension in the plates. All plates were incubated at 37°C for 24 h. The diameter of inhibition zone created by each disc was measured (in mm) using a micrometer.

### Data analysis

The inhibition zone data were expressed as means ± standard error. The data were subjected to ANOVA, followed by Duncan’s post hoc test to evaluate significant differences among the groups of treatments. Meanwhile, the comparison between bacteria in each concentration was performed using a t-test. All significant tests were at *P<*0.05 levels and all analysis was done using SPSS 22 (SPSS, Inc., USA). The data of the phytochemical content and the concentration of flavonoid and phenolic were analyzed descriptively.

## Results and discussion

**Figure 1.**
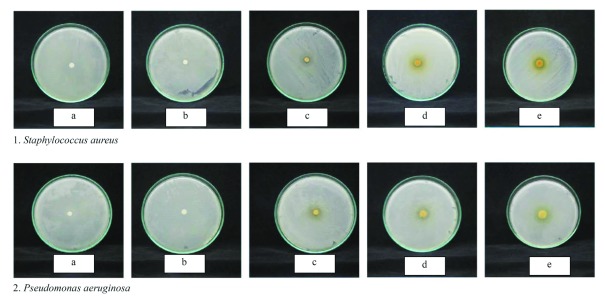
Inhibition zone of *Terminalia catappa* brown leaves ethanolic extract against *Staphylococcus aureus* and *Pseudomonas aeruginosa*. ( **a**) Negative control, ( **b**) positive control (0.1% gentamycin sulfate), *Terminalia catappa* extract ( **c**) 30%, ( **d**) 60%, ( **e**) 90%. Images shown are representative of n=3 repeats.

The dried extract of *T. catappa* brown leaves contained flavonoids, quinon, phenolics, triterpenoids, and tannins. There were no alkaloids, steroids or saponin found in the dried extract. Total phenolic (208.722 mg gallic acid equivalent/g extract) and total flavonoid (35.7671 mg quercetin equivalent/g extract) were detected in the dried extract. The inhibition zone diameters of ethanolic extracts ranged from 1.73 to 9.06 mm for *S. aureus*, and from 1.83 to 6.5 mm for *P. aeruginosa*. Increasing the extract concentration increased the inhibition zone diameters for both bacteria ( Figure 1). *P. aeruginosa* was more resistant to high concentrations of extract (90%) than *S. aureus* ( Table 1). According to Xie *et al*. ^23^, flavonoids are known antibacterial agents against a wide range of pathogenic bacteria. In addition, Fu *et al*. ^24^ also revealed that phenolic extracts from some plants also have antibacterial effects against many kinds of bacteria. The data showing the inhibition zone diameters for both bacteria at each concentration of extract can be seen in Dataset 1 ^25^.

Inhibition zone diameters for both bacteria at different concentration of extracts and images of every repeat experiment performed Click here for additional data file. Copyright: © 2018 Allyn OQ et al. 2018 Data associated with the article are available under the terms of the Creative Commons Zero "No rights reserved" data waiver (CC0 1.0 Public domain dedication).

**Table 1.** Inhibitory zone diameter (in mm) of *Staphylococcus aureus* and *Pseudomonas aeruginosa* after treated with different concentration of brown leaves of *T. catappa* ethanolic extract.

Bacteria	Positive control	Extract concentration		
		30%	60%	90%
*Staphylococcus aureus*	5.78±0,27 ^a,1^	1.73±0,24 ^b,1^	5.28±1,06 ^a,c,1^	9.06±0,56 ^d,1^
*Pseudomonas aeruginosa*	7.15±0,20 ^a,2^	1.83±0,87 ^b,1^	4.53±0,78 ^c,1^	6.50±0,13 ^a,d,2^

Different superscript letters in the same row indicate significantly different mean values for different treatments at *P*<0.05. Different superscript numbers in the same column indicate significantly different mean values for different treatments at *P*<0.05. The negative control was omitted as no inhibition zone was present. Positive control, 0.1% gentamycin sulfate.

## Conclusion

Ethanolic extracts of the brown leaves of *T. catappa* have potential antibacterial effects against *S. aureus* and *P. aeruginosa*, indicated by the inhibition zone formed around the extract. The inhibition zone diameter increased with increasing concentrations of *T. catappa* extract. *P. aeruginosa* exhibited more resistance to high concentrations of ethanol extracts of the brown leaves of *T. catappa* than *S. aureus*.

## Data availability

The data referenced by this article are under copyright with the following copyright statement: Copyright: © 2018 Allyn OQ et al.

Data associated with the article are available under the terms of the Creative Commons Zero "No rights reserved" data waiver (CC0 1.0 Public domain dedication). 

Dataset 1. Inhibition zone diameters for both bacteria at different concentration of extracts and images of every repeat experiment performed. DOI: https://doi.org/10.5256/f1000research.15998.d215169 ^25^.
