# Supplementary material for: Antimicrobial activity of Terminalia catappa brown leaf extracts against Staphylococcus aureus ATCC 25923 and Pseudomonas aeruginosa ATCC 27853
Source: F1000Res. 2018 Sep 4;7:1406. [Version 1] doi: 10.12688/f1000research.15998.1 (PMC6206604; doi:10.12688/f1000research.15998.1)
Supplement: Peer review history

# Peer review: Referee response for version 1

**DOI:** 10.5256/f1000research.17473.r37962

**Published:** 2018-10-23

**Authors:** Cimi Ilmiawati

**Affiliations:** 1 Division of Environmental Toxicology, Department of Pharmacology, Faculty of Medicine, Andalas University, Padang, Indonesia

Comments:

 This study contributes information on the potential of T. catappa leaves extract as an antibacterial alternative to address antibacterial resistance problems in fish farming.

Methods

 Site and time: Please specify the affiliation of the laboratory (whether it belongs to a department in a university or else).

Plant materials

 Please correct the writing of Mulawarman University.

 How to preserve the crushed leaves? How long the preservation until the extraction?

Phytochemical contents

 What is the purpose of qualitative and quantitave phytochemical analysis in relation to the study objectives? Please elaborate in the introduction part. 

Results

1. The inhibition zone in Figure 1 for the positive control is not very clear, therefore a zoom in figure is required to judge the result.
2. I was expecting to see three discs in each plate of treatment and this experiment shall be replicated three times, before analyzing the data and arriving into a conclusion. Please comment on your replication method.    

I have read this submission. I believe that I have an appropriate level of expertise to confirm that it is of an acceptable scientific standard.

# Peer review: Referee response for version 1

**DOI:** 10.5256/f1000research.17473.r37961

**Published:** 2018-10-23

**Authors:** Md Fakruddin

**Affiliations:** 1 Laboratory of stem cell stress, International Research Center for Medical Sciences, Kumamoto University, Kumamoto , Japan

1. In the title, it should be mention ethanol extract, as the authors only evaluate ethanol extract of the leaves.
2. In the figure 1, the quality is poor, so it seems the positive control do not have any inhibition zone!
3. Only one strain of each bacteria was tested, it would be more authentic if more types of strains as well as some wild or environmental strains were tested.
4. MIC/MBC of the extract against these bacteria should be determined. 
5. Data of this study should be compared with similar data published already. It is understood, that there may be no data of this particular species, but other species of terminalia can be consulted.

I have read this submission. I believe that I have an appropriate level of expertise to state that I do not consider it to be of an acceptable scientific standard, for reasons outlined above.

# Peer review: Referee response for version 1

**DOI:** 10.5256/f1000research.17473.r37963

**Published:** 2018-09-20

**Authors:** Edwin Setiawan

**Affiliations:** 1 Department of Biology, Faculty of Natural Sciences, Sepuluh Nopember Institute of Technology, Surabaya, Indonesia

The study is designed appropriately and and technical procedure has been taken sufficiently to answer the research question. Furthermore, methods and analyses process also sufficient to be replicated by readers. In addition, statistical method that used is also appropriate. They author need to highlighted and emphasized on the results and discussion with expanding their discussion with comparing another result that similar to this study.

 In the result and discussion part, it is better to compare phenolic and flavonoid content of Terminalia catappa to other plant extract that close to Terminalia catappa if possible. Or in other words, comparing other similar research that used plant bioactive compound for antibacterial to research. Therefore, this result and discussion part could be expanded comprehensively.

 In addition, simple and short explanation on how the mechanism flavonoid and phenolic inhibit bacterial growth can be added in this part.

I have read this submission. I believe that I have an appropriate level of expertise to confirm that it is of an acceptable scientific standard.
